# Decellularization in Tissue Engineering and Regenerative Medicine: Evaluation, Modification, and Application Methods

**DOI:** 10.3389/fbioe.2022.805299

**Published:** 2022-04-25

**Authors:** Afarin Neishabouri, Alireza Soltani Khaboushan, Faezeh Daghigh, Abdol-Mohammad Kajbafzadeh, Masoumeh Majidi Zolbin

**Affiliations:** ^1^ Pediatric Urology and Regenerative Medicine Research Center, Children’s Medical Center, Pediatric Center of Excellence, Tehran University of Medical Science, Tehran, Iran; ^2^ Students’ Scientific Research Center, Tehran University of Medical Sciences, Tehran, Iran; ^3^ Department of Physiology, Faculty of Medicine, Tabriz Medical Sciences, Islamic Azad University, Tabriz, Iran

**Keywords:** extracellular matrix, recellularization, regenerative medicine, decellularizalion, tissue engineering

## Abstract

Reproduction of different tissues using scaffolds and materials is a major element in regenerative medicine. The regeneration of whole organs with decellularized extracellular matrix (dECM) has remained a goal despite the use of these materials for different purposes. Recently, decellularization techniques have been widely used in producing scaffolds that are appropriate for regenerating damaged organs and may be able to overcome the shortage of donor organs. Decellularized ECM offers several advantages over synthetic compounds, including the preserved natural microenvironment features. Different decellularization methods have been developed, each of which is appropriate for removing cells from specific tissues under certain conditions. A variety of methods have been advanced for evaluating the decellularization process in terms of cell removal efficiency, tissue ultrastructure preservation, toxicity, biocompatibility, biodegradability, and mechanical resistance in order to enhance the efficacy of decellularization methods. Modification techniques improve the characteristics of decellularized scaffolds, making them available for the regeneration of damaged tissues. Moreover, modification of scaffolds makes them appropriate options for drug delivery, disease modeling, and improving stem cells growth and proliferation. However, considering different challenges in the way of decellularization methods and application of decellularized scaffolds, this field is constantly developing and progressively moving forward. This review has outlined recent decellularization and sterilization strategies, evaluation tests for efficient decellularization, materials processing, application, and challenges and future outlooks of decellularization in regenerative medicine and tissue engineering.

## 1 Introduction

Tissue engineering, as a division of regenerative medicine, combines engineering and biological science in order to reproduce tissues and organs that can help to overcome the lack of enough donor organs (Shafiee and Atala, 2017). Tissue engineering applies cells into desirable biological structures in a defined framework to restore the normal function of tissues. This process includes three cornerstones, namely scaffolds, cells, and signaling factors. As a critical component in tissue engineering, scaffolds provide mechanical stability and structural support for exogenous cell attachment and proliferation and facilitate the delivery of required growth factors for tissue regeneration (Vacanti and Langer, 1999; Soltani Khaboushan et al., 2021).

Scaffolds could be made up of natural tissues harvested from animal or human sources or being constructed using synthetic biomaterials. Natural scaffolds represent biological characteristics that better fit the regular tissue microenvironment, promoting appropriate cellular interactions, biocompatibility, and degradability. Decellularization is the process of eliminating cells and their components (especially DNA and RNA) from the extracellular matrix (ECM) to yield a natural matrix with saved mechanical integrity. It has been demonstrated that decellularized extracellular matrix (dECM) is a suitable type of natural scaffold for tissue engineering since the ECM plays a crucial role in tissue development (Gilbert et al., 2006).

ECM is mainly composed of water, proteins (mainly collagen), and polysaccharides (Frantz et al., 2010). The composition and arrangement of ECM components and microenvironmental conditions (e.g., mechanical properties, pH, CO_2_ concentration) of the matrix differ from one tissue to another based on the tissue function and its residing cells that secrete ECM components (Kawecki et al., 2018). Fibroblasts, adipocytes, and chondrocytes are among the cells involved in the formation of ECM components (including growth factors and structural proteins such as fibronectin). Physical, chemical, and biological methods have been utilized to produce acellular scaffolds, either by perfusion- or immersion/agitation-based systems, or even a combination of methods (Crapo et al., 2011).

So far, a wide variety of decellularization methods have been studied and developed (Crapo et al., 2011; Fu et al., 2014; Keane et al., 2015; Choudhury et al., 2020). Since acellular scaffolds have low immunogenicity and are biologically recognizable, they are beneficial for cell adhesion, proliferation, and survival. Because of these properties, decellularized materials have the potential to regenerate injured tissues or organs (Damodaran and Vermette, 2018). After decellularization, dECM should be sterilized, and several tests should assess the efficacy of decellularization. These tests are carried out to ensure the removal of cellular contents and preservation of biochemical and mechanical properties and include macroscopic and microscopic assessments, staining for evaluation of remaining components, and mechanical analyzes (Crapo et al., 2011). Moreover, these acellular scaffolds can be processed before application to acquire desired characteristics of the ECM for further use. Modifying and refining the dECM, recellularizing the scaffold, and providing a suitable biochemical and physical environment via bioreactors, could improve its characteristics and boost its regeneration ability in host tissues (Garreta et al., 2017).

The application of dECM as wound healing products and surgical mesh devices has been reported (Damodaran and Vermette, 2018; Daryabari et al., 2019; Uday Chandrika et al., 2021). In addition to therapeutic applications, dECM could be used for modeling various diseases, such as tumors, which helps to understand the pathophysiology and progression process of the disease (Liu et al., 2019; Pina et al., 2019). Despite the recent progress, tissue engineering is still in its early stages, and multiple studies are being conducted to develop functional organs. Up to the present, research was mainly directed towards discovering the ideal decellularization methods; and translational studies were performed to achieve functioning materials. This review aims to provide an overview of decellularization techniques, evaluation tests, dECM modification, practical approaches, clinical application, current insufficiencies, and future prospects in tissue decellularization.

## 2 Decellularization Agents and Methods

Several decellularization techniques have been developed to date to reconstruct different types of living organs. The main principle in all methods is removing cellular material and leaving the ECM ultrastructure unchanged in the tissue. Decellularization techniques differ in terms of applied materials (reagent combinations) and the routes used to deliver the main reagent, namely vascular, airway, or both (Uygun et al., 2010; Badylak et al., 2011; Crapo et al., 2011; Daryabari et al., 2019).

Generally, tissue decellularization methods are classified into three main groups: chemical methods, such as alkaline/acid, detergents, and alcohols; physical methods, such as electroporation, pressurization, freeze/thaw; biological methods, such as enzymes (Table 1) (Badylak et al., 2011; Gilpin and Yang, 2017). Several characteristics could sway the quality of the tissue decellularization process, including cell density, matrix thickness, and tissue morphology. These characteristics are different in various tissues; Thus, it is crucial to determine which method is the most suitable one for a specific tissue (Crapo et al., 2011; Heath, 2019).

**TABLE 1:** Decellularization methods and agents.

Method	Category	Agent	Properties	References
Chemical	Organic Solvents	Alcohols (e.g., ethanol)	Cell lysis by dehydrating the tissue	Wollmann et al. (2019), Walawalkar and Almelkar, (2019)
		Acetone	May disrupt ECM ultrastructure	
		TnBP	Disrupts protein-protein connections	Deeken et al. (2011), Duarte et al. (2021)
			May enhance collagen crosslinking	
	Chelators	EDTA	Disrupts cell adhesions	Rieder et al. (2004), Cheng et al. (2019), Caralt et al. (2015), Huh et al. (2018)
		EGTA	Usually combined with other agents such as trypsin due to low efficacy in cell removal	
	Toxins	Latrunculin B	Acts through actin rearrangement	Reyna et al. (2020), Assmann et al. (2017)
			Mostly used for decellularizing skeletal muscle	
	Ionic detergents	SDS	High efficacy in cell removal by solubilizing cell membrane	Hudson et al. (2004), Alshaikh et al. (2019), O'Neill et al. (2013)
		SD		
		Triton X-200	May disrupt ECM ultrastructure	
	Non-ionic detergents	Triton X-100	Gentle cell removal by disrupting lipid-lipid and lipid-protein connections	Fu et al. (2010), Huh et al. (2018), Liao et al. (2008)
			Gentle disruption of the ECM structure	
	Zwitterionic detergents	CHAPS	Properties of ionic and non-ionic detergents May disrupt basement membrane	Tsuchiya et al. (2014), Gupta et al. (2017), Song et al. (2021)
		SB-10		
		SB-16		
	Hypotonic and hypertonic solutions	Sodium chloride solution	Osmotic shock induction	Kim et al. (2016), Goktas et al. (2014)
			Low efficacy in remnant removal	
			Minimal ECM disruption	
	Acid and Alkaline	PAA and EDTA Sodium hydroxide	Solubilizing cytoplasmic cell components and nucleic acid disruption Can disrupt ECM components	Wollmann et al. (2019), Poornejad et al. (2016), Mimler et al. (2019)
Biological	Esterase	Phospholipase A2	Cleaving ester bonds in the cell membrane May disrupt ECM components	Isidan et al. (2019), Huang et al. (2011)
	Protease	Trypsin	Cleaving peptide bonds May disrupt ECM components over prolonged exposure	Prasertsung et al. (2008), Rahman et al. (2018), Pouliot et al. (2020)
		Pepsin		
		Dispase		
	Nuclease	DNase	Cleaving nucleotide bonds	Simsa et al. (2018), McCrary et al. (2020b), Philips et al. (2018b)
		RNase		
Physical	Freeze-thaw cycles		Cell death due to crystallization of intracellular water Extracellular crystallization can disrupt ECM	Roth et al. (2017), Fernández-Pérez and Ahearne, (2019b)
	Agitation		Cell death directly or by assisting in chemical exposure and remnant/toxic removal	Starnecker et al. (2018), Simsa et al. (2019), Yusof et al. (2019)
	Pressure		Cell Membrane destruction May damage biomechanical properties of ECM	Funamoto et al. (2010), Watanabe et al. (2019)
	Supercritical fluids		Assisting in chemical exposure and remnant/toxic removal	Chou et al. (2020), Duarte et al. (2021)

Abbreviations: TnBP, Tri(n-butyl) phosphate; EDTA, Ethylene diamine tetraacetic acid; EGTA, Ethylene glycol tetraacetic acid; SDS, Sodium dodecyl sulfate; SD, Sodium deoxycholate; CHAPS, 3-[(3-cholamidopropyl)dimethylammonio]-1-propanesulfonate; SB, Sulfobetaine; PAA, Peracetic acid; DNase, Deoxyribonuclease; RNase, Ribonuclease.

### 2.1 Chemical Agents

#### 2.1.1 Ionic Detergents

Ionic detergents act by solubilizing DNA and cell membrane and tend to denature the proteins, thus decreasing collagen integrity. Ionic detergents are powerful in removing glycosaminoglycans (GAGs) and growth factors, therefore destroying ECM rigidity and function. Sodium dodecyl sulfate (SDS), sodium deoxycholate (SD), and Triton X-200 are ionic detergents that have been used in decellularization (Hudson et al., 2004; Gilbert et al., 2006; Lumpkins et al., 2008; Montoya and McFetridge, 2009; Zhou et al., 2010). Alshaikh et al. (Alshaikh et al., 2019) have examined SDS and SD in ovary decellularization. They have suggested a better ECM preservation in SD than SDS but lower donor DNA content in SDS. Due to polarity, ionic detergents such as SDS are hard to remove from ECM, and extensive wash with non-ionic detergents such as Triton X-100 is usually needed to take away the remnant ionic detergents from tissue (Gilpin and Yang, 2017).

#### 2.1.2 Non-Ionic Detergents

Non-ionic detergents, such as Triton X-100, can strongly break lipid-lipid and lipid-protein bonds, but they are less effective on protein-protein interaction. Although they maintain the ultrastructure of the decellularized tissue and preserve the growth factors, they are less effective in removing cellular materials than SDS. The Triton X-100 is not appropriate for tissues where GAGs and lipids are important parts. Accordingly, the effectiveness of non-ionic detergents depends on the tissue undergoing the decellularization process (Gilbert et al., 2006; Lumpkins et al., 2008; Fu et al., 2010; Crapo et al., 2011; Gupta et al., 2018).

#### 2.1.3 Zwitterionic Detergents

Zwitterionic detergents have the properties of both ionic and non-ionic detergents. They have shown better cell removal than non-ionic detergents and improved preservation of the ECM ultrastructure than ionic detergents (Hudson et al., 2004; Gupta et al., 2018; Heath, 2019). Zwitterionic detergents, include sulfobetaine-10 (SB-10), SB-16, Tri (n-butyl) phosphate (TnBP), and 3-[(3-cholamidopropyl) dimethylammonio]-1-propanesulfonate (CHAPS). CHAPS is a common zwitterionic agent that has been widely used in tissue decellularization (Tsuchiya et al., 2016; Gupta et al., 2018). TnBP is an organic solvent that dissociates protein-protein Interactions. TnBP was reported to match SDS in cell removal in tendon and ligament tissues (Deeken et al., 2011). Kuna et al. (Kuna et al., 2018) have reported that decellularization of the human saphenous vein using Triton X-100, TnBP, and deoxyribonuclease (DNase) resulted in properly removing the cells.

#### 2.1.4 Chelators and Toxins

Ethylene glycol tetraacetic acid (EGTA) and ethylenediaminetetraacetic acid (EDTA) are used as chelating agents in organ decellularization through binding to divalent metal cations at the cell adhesion site of ECM. This binding was found to dissociate the cell from the remaining ECM. Due to their ability to damage cells, Cytotoxic agents could be used in the decellularization process (Crapo et al., 2011). Reyna et al. (Reyna et al., 2020) have shown the high efficiency of latrunculin, a toxin, in removing skeletal muscle cells by disrupting actin and myosin, thus damaging the cell and reducing DNA content to less than 10% compared to controls have shown the high efficiency of latrunculin, a toxin, in removing skeletal muscle cells by reducing DNA content to less than 10% compared to controls. They stated that this toxin is more effective in decellularizing skeletal muscle than ionic and non-ionic agent treatments.

#### 2.1.5 Bases

Solutions with extreme pH were indicated to be effective in the decellularization process. Alkaline substances including sodium sulfide, ammonium hydroxide, calcium hydroxide, and sodium hydroxide were suggested as commonly used in organ decellularization processes. It has been shown that increasing the PH of CHAPS during decellularization increases the effectiveness of cell and protein removal. Bases could eliminate growth factors and disrupt the mechanical structure of the scaffold (Komai and Ushiki, 1991; Prasertsung et al., 2008; Choi et al., 2010; Reing et al., 2010; Sheridan et al., 2012; Tsuchiya et al., 2014; Gupta et al., 2018).

#### 2.1.6 Acids

Acids were found to dissociate nuclear DNA from ECM by disrupting nucleic acids and solubilizing cytoplasmic components. Moreover, acids could facilitate the denaturation of the biomolecules. Acetic acid, hydrochloric acid, and sulfuric acid can disrupt cell membranes and be used in decellularization (Gilbert et al., 2006; Gupta et al., 2018). Lin et al. used and compared formic acid, acetic acid, and citric acid for porcine cornea decellularization (Lin et al., 2019). They showed that formic acid treatment had the optimal decellularizing effect and preserved *in vitro* and *in vivo* recellularization. Peracetic acid (PAA) can also be used as a decellularizing agent; however, it is mostly used as a sterilizing agent and has an unsuitable impact on decellularization. Kao and colleagues (Kao et al., 2020) have reported that the use of PAA in bladder decellularization was not successful because the levels of remaining DNA were similar to the native tissue.

#### 2.1.7 Alcohols

Alcohols play their role in the decellularization process via dehydration; they diffuse into the cells, replace the intracellular water, disrupt the cells, and decrease their genetic materials. Alcohols such as methanol and ethanol are effective in lipid solubilization. Due to their role in tissue fixation and protein deposition, ethanol and methanol may affect the ultrastructure of the tissues. Lumpkin et al. have demonstrated that the use of acetone/ethanol in the decellularization of the temporomandibular joint disc came up with a stiffer tissue compared to Triton X-100 and SDS, and the mechanical characteristics of the decellularized tissue were not appropriately preserved (Lumpkins et al., 2008; Jamur and Oliver, 2010; Crapo et al., 2011).

#### 2.1.8 Hypertonic and Hypotonic Solutions

Hypotonic and hypertonic solutions can lead to cell lysis and disruption of the DNA. Although they do not remove the cellular debris, they could improve decellularization in combination with other chemical reagents since they do not disturb ECM composition (Woods and Gratzer, 2005; Gupta et al., 2018).

### 2.2 Biological Enzymes

Enzymatic agents include phospholipase A2, proteases (e.g., trypsin, dispase), and nucleases (e.g., DNase, ribonuclease (RNase) are used in the decellularization process. Trypsin breaks peptides containing Lys and Arg, and in extended exposure, it may damage the ECM structure (Stenn et al., 1989; Gilbert et al., 2006; Rahman et al., 2018). Dispase chiefly cleaves fibronectin and collagen IV. It has been used in the decellularization of the porcine cornea and skin (Wilson et al., 2016; Joszko et al., 2019). Nucleases are mainly used in combination with other detergents to expedite the removal of DNAs and RNAs from the scaffold (Grauss et al., 2005; Heath, 2019). The application of phospholipase A2 in decellularization helps maintain collagen and proteoglycans in the tissue. It has been demonstrated that phospholipase A2, along with SD, was influential in producing the acellular porcine corneal stroma (Wu et al., 2009; Rahman et al., 2018).

### 2.3 Physical Methods

#### 2.3.1 Freeze-Thaw Cycles

The freeze-thaw cycle is being done by fluctuation between freezing temperature (−80c°) and biological temperature. The freeze-thaw cycle disrupts the cell membranes and cell lysis via the formation of intracellular crystals. It has been demonstrated that this method considerably keeps the structure of the ECM but does not effectively remove the remnant cellular debris; thus, further detergents are needed after the freeze-thaw cycle. Multiple freeze-thaw cycles can be used in the decellularization process, whereas they could deteriorate the ECM structure (Pulver et al., 2014; Xing et al., 2015; Rahman et al., 2018).

#### 2.3.2 Agitation Immersion and Pressure

The agitation in conjunction with immersion leads to cell lysis, but it is mostly used with chemical reagents to enhance the exposure of the ECM to the detergents and to augment the decellularization process. Agitation could be applied in the decellularization of the thin tissues, including the small intestine and bladder. In addition, the use of agitation and immersion in the decellularization of the tracheal tissue has been reported. The time of the protocol and intensity of agitation depends on the thickness of the tissue. Agitation may cause cell lysis before exposure to the detergents; thus, deteriorating the structure of the ECM. Furthermore, pressure could promote accessibility of the detergents to the ECM, hence decreasing the time of the decellularization process. Also, it can improve the removal of cellular remnant materials. The pressure leads to minimal changes in ECM structure, even less than agitation (Crapo et al., 2011; Heath, 2019; Rabbani et al., 2021). Sonication is another physical method and can be considered a subtype of agitation. It has been demonstrated that direct or indirect sonication increases agent penetration to the scaffold, therefore enhancing chemical decellularization while causing less damage to the ECM structural content (Forouzesh et al., 2019).

#### 2.3.3 Supercritical Fluids

The supercritical fluids are over their critical pressure and temperature where it is not distinctly gas or liquid. Due to high permeability, these fluids could easily be removed from the tissue without the need for further washing. They can remove remnant cellular particles from the scaffold and decrease harmful changes in the ECM. Supercritical carbon dioxide has recently attracted attention in tissue decellularization because its critical temperature is appropriate for ECM processing. Recently, different studies reported complete effective removal of porcine skin via supercritical CO_2_ (Crapo et al., 2011; Chou et al., 2020; Rabbani et al., 2021). Supercritical CO_2_ not only decellularizes different tissues with better ECM structure preservation compared to conventional detergent-based techniques but also is used for sterilization of the dECM. Efficient cell removal and preserved ECM integrity were achieved by applying different supercritical CO_2_-based protocols on the optic nerve, myocardium, and cornea (Topuz et al., 2020).

## 3 Sterilization

Decontamination of the dECM via sterilization and disinfection process is necessary before further evaluations and *in vitro* or *in vivo* application. Sterilization kills all microorganisms, but the disinfection process only removes vegetative microorganisms and does not affect the bacterial spores. There are different sterilization methods that can be used for dECM methods. Appropriate sterilization and disinfection method should be selected considering various properties of the decellularized scaffold, including chemical and physical characteristics. Moreover, the needed time, availability, and target of application are important factors in choosing the proper method for sterilization (Kajbafzadeh et al., 2013; Fidalgo et al., 2018; Moradi et al., 2020a; Tao et al., 2021). Irradiation is a physical sterilization strategy that directly destroys nucleic acids and proteins from microorganisms. Gamma irradiation is extensively used for the sterilization of various tissues. Gamma irradiation can effectively sterilize corneal xenografts while preserving their structure and integrity. The irradiation has strong penetrance to the tissue without toxicity. Ultraviolet (UV) rays as a convenient physical disinfectant for the environment and surfaces are used for disinfecting thin decellularized scaffolds with a large surface. Large tissues such as decellularized kidneys cannot entirely be sterilized by UV, and it may interfere with further cell seeding on dECM (Islam et al., 2019; Moradi et al., 2020a; Gosztyla et al., 2020; Tao et al., 2021).

Ethylene oxide sterilizes the tissues by disrupting the function of the nucleic acids and proteins of the microorganisms. With its strong penetration, ethylene oxide can be used for sterilizing various decellularized scaffolds without causing toxicity. Peroxides are widely used as a disinfectant, which can also cause sterilization in specific conditions. They do not produce any toxic product after decomposition (Hennessy et al., 2017; Moradi et al., 2020a; Tao et al., 2021). According to a study, it has been demonstrated that PAA, as a peroxide, has the ability to decontaminate acellular rabbit kidney completely, whereas γ-irradiation destructed the structure tissue and UV failed to eliminate microorganisms (Moradi et al., 2020a). Alcohols are disinfectants that destroy the proteins within microorganisms, but they do not remove spores (Tao et al., 2021). They have minimal effects on the tissue structure; thus, they have been widely used to disinfect dECM derived from various tissues. Supercritical CO_2_, in addition to its effects on decellularization process, can lead to disinfection and sterilization of the decellularized scaffolds (Hennessy et al., 2017; Antons et al., 2018; Tao et al., 2021). CO_2_ laser, although less commonly used, has a great potential to be used for the sterilization process. CO_2_ laser bursts effectively removed bacteria inoculated on pig skin in an experiment. A sterile environment is a prerequisite of regenerative endodontic treatment, which could be effectively achieved with the assistance of a CO_2_ laser (Mullarky et al., 1985; Nammour and Majerus, 1991; Divya et al., 2021). Low-level laser with 660 nm wavelength and 100 mW power has been used for sterilization of the decellularized lung tissues (Lopes Guimarães et al., 2020). Laser is an appropriate option for effectively disinfecting tissues, and its potential for disinfecting the dECM should be further explored (Mullarky et al., 1985; Nammour and Majerus, 1991; Divya et al., 2021).

These methods may impact the structural and biochemical characteristics of the matrices. The γ-irradiation mainly affects the ultrastructure and mechanical properties of the scaffold, while ethanol and peracetic acid increase the ECM crosslinking (Hennessy et al., 2017; Johnson et al., 2017; Islam et al., 2019). In order to assess the efficacy of the sterilization method and its effects on the scaffold, several evaluations, including histological evaluations, 3-(4, 5-dimethylthiazol-2-yl)-2, 5-diphenyltetrazolium bromide (MTT) assay, mechanical tests, and bacterial and fungal cultures may be applied (Moradi et al., 2020b).

## 4 Evaluation of Decellularized Extracellular Matrix

**FIGURE 1:**
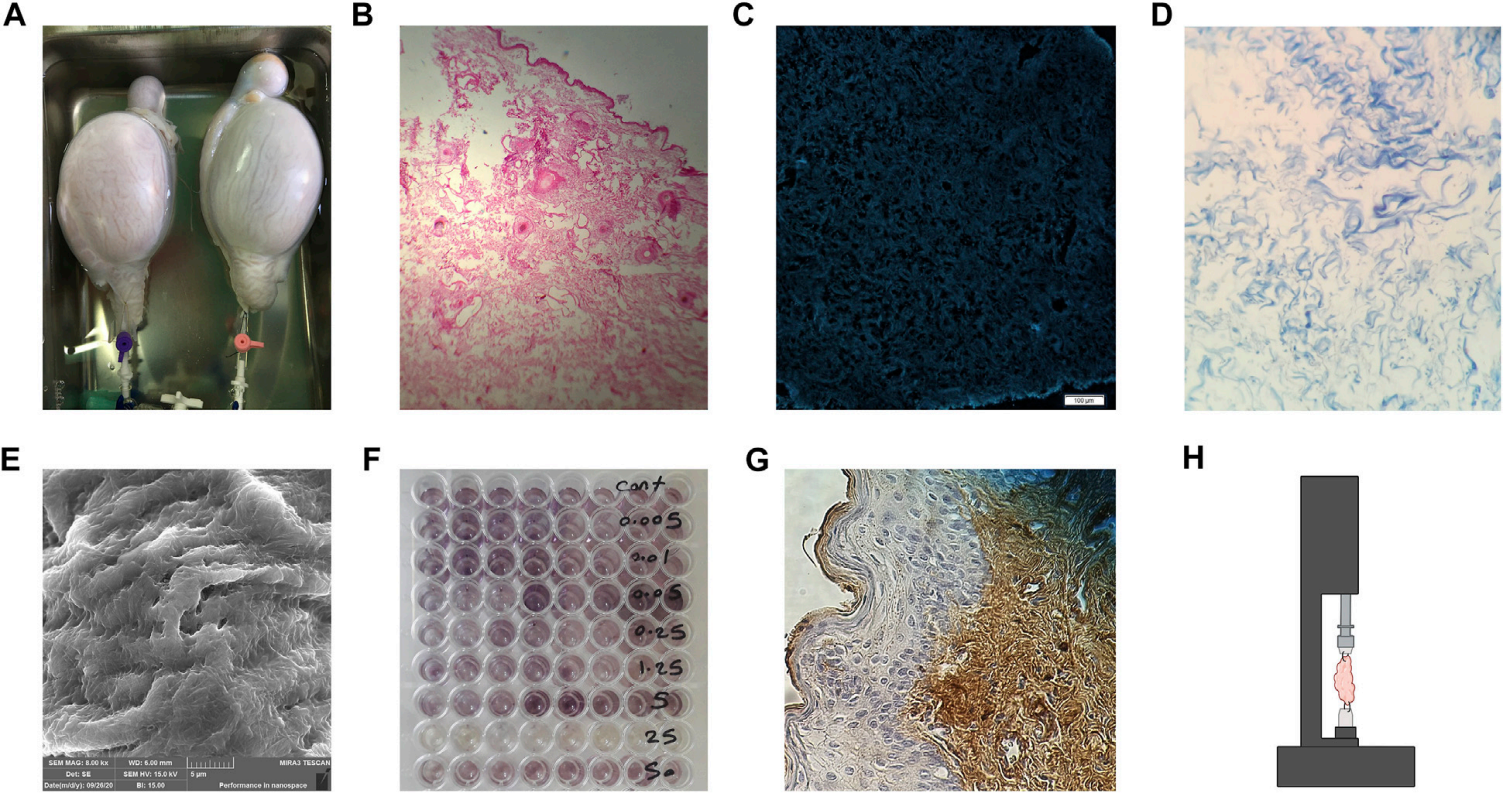
Evaluating the dECM. **(A)** Gross inspection of the decellularized ovine whole testes. **(B)** Hematoxylin and Eosin H&E staining of decellularized human breast skin, no nucleus can be observed. **(C)** 4’,6-diamidino-2-phenylindole (DAPI) staining of decellularized human skin with no stained DNA. **(D)** Masson’s Trichome staining of the human ovary, showing preserved collagen fibers while no nucleus is seen. **(E)** SEM imaging of decellularized ovine ovary tissue. **(F)** 3-(4, 5-dimethylthiazol-2-yl)-2, 5-diphenyltetrazolium bromide (MTT) assay, showing cellular viability and activity where the purple dye is observed. **(G)** Immunohistochemistry (IHC) staining using antibodies against collagen I in human skin after recellularization. **(H)** Schematic imaging of the tensile test, tissue is placed in the middle of the apparatus and dragged in opposite directions to evaluate the mechanical strength of the dECM.

Although decellularization techniques cannot remove 100% of cell material, quantitative analysis of cell components such as mitochondria, double-stranded DNA (dsDNA), and membrane-associated molecules (e.g., phospholipids) is a crucial way to ensure the effectiveness of the decellularization model. On the other hand, the ideal decellularization method should conserve biochemical and mechanical properties with the lowest toxicity rate for the subsequent recellularizing phase to occur. Various evaluation tests and assessments have been introduced to appraise the different characteristics of the dECM (Figure 1).

### 4.1 Macroscopic Evaluation

Despite the fact that gross inspection alone is not an entirely reliable evaluation method, it is the first step in the evaluation of the decellularized scaffold, which should be followed by more accurate assessments. Different studies use macroscopic imaging analysis as one of the first assessment steps to determine the level of transparency of dECM compared to native tissue (Philips et al., 2018a). Moreover, light transmittance is essential in several organ reconstructions, such as cornea (Oliveira et al., 2013). Transparency is measured by placing samples on a patterned surface. Then, the intensity is calculated by assessing the transmitted light by processing images with software (Oliveira et al., 2013; Philips et al., 2018a; Fernández-Pérez and Ahearne, 2019a).

### 4.2 Cell Removal Efficacy and Immunogenicity

Various tests assay the effectiveness of the decellularization method. Complete removal of antigens and nucleic acids, along with other cellular components, ensures the biocompatibility of the scaffold. The dECM must be implanted without host immune response activation and graft rejection or the formation of vascular thrombosis. Besides, transgenic transmission has been reported by retained DNA in genetically engineered tissue implantation (Hussein et al., 2016). Crapo et al. have suggested criteria for decellularization satisfaction with cut-off amounts for dsDNA per mg ECM dry weight and DNA fragment length, 50ng and 20bp, respectively, and lack of visible nuclear content in microscopic evaluation (Crapo et al., 2011). Also, extended agent exposure has been used to reduce DNA content by approximately 93% (Tenreiro et al., 2021). Histological analysis of the tissue allows evaluation of general morphology of dECM, as well as cell content and structural integrity (Philips et al., 2018a). Hematoxylin and Eosin (H&E) is mainly used in assessing tissue morphology and cell nuclei, while fluorescent staining with 4’,6-diamidino-2-phenylindole (DAPI) are used in the detection of remaining nuclear structures (Crapo et al., 2011; Su et al., 2018). Other histological staining such as Safrin O, Movat’s pentachrome, Masson’s Trichome, and Hoescht can also be used to evaluate the existence of remnant DNA or cytoplasmic and extracellular molecules in decellularized tissue (Garreta et al., 2017; Gaetani et al., 2018).

DNA quantification using immunofluorescence, electrophoresis, or polymerase chain reaction is also of great use (Pors et al., 2019; Naik et al., 2020; Hussein et al., 2018; da Mata Martins et al., 2020). Of note, electron microscopic assessment can be but is not commonly used to evaluate the existence of nuclear material or cytoplasmic debris due to the cost and the technical complexity (Gilbert et al., 2006).

### 4.3 Ultrastructure Evaluation

Preservation of 3 dimensional (3D) architecture and structure of the scaffold can be evaluated using different assessments, such as electron microscope (Giraldo-Gomez et al., 2019). A scanning electron microscope (SEM) is used to reveal surface topography, while transmission electron microscopy (TEM) is used for the detailed orientation of materials and cellular organelles (Márquez et al., 2009). These assessments are utilized to indicate the efficacy of decellularization treatment in conserving the native structure of ECM. In addition, they can show the debris and remaining cell components in the decellularized tissue (Philips et al., 2018a). In a study by Forouzesh et al., SEM images showed sonication-induced micropores with 0.5–5 μm diameter on the surface of decellularized cartilage tissue. Compared to the ultrasound-induced ones, these smaller micropores resulted in increased surface roughness, and suitable cell adhesion was imaged by SEM (Forouzesh et al., 2019).

### 4.4 Cytocompatibility

Prior to *in vivo* use, it is crucial to investigate the cellular interaction of cells with the decellularized scaffold. In other words, the damaging effects of the decellularization method or agent on the scaffolds should be assessed *ex vivo* to guarantee the clinical efficacy of the dECM. In this regard, the initial step is to quantify the remaining chemicals after washing and sterilizing. Decellularizing and sterilizing agents are cytotoxic and prevent efficient recellularization. The threshold for chemicals cytotoxic concentration, such as SDS has been investigated (Zvarova et al., 2016). The most valued biocompatibility test is *in vitro* cell culture, either indirect or direct (Hussein et al., 2016). In indirect contact assay, cellular proliferation (e.g., live/dead assay or DNA quantification) and metabolic activity (e.g., MTT assay) will be evaluated when cultured in samples extraction, while in direct contact assay, cells will be directly cultured on the decellularized samples and investigated.

### 4.5 Biochemical Analysis

Following decellularization, it is necessary to determine the remaining desirable ECM components such as GAGs, elastic fibers, collagens, and adhesion proteins like fibronectin and laminin in the decellularized tissue (Gilbert et al., 2006). An adequate amount of these molecules in ECM contributes to the tissue’s normal function, structure, and mechanical characteristics. Various stains, kits, and immunohistochemistry (IHC) markers can be used for the detection of these materials (Garreta et al., 2017; Kajbafzadeh et al., 2019). For instance, Masson’s trichrome is used for collagen fiber staining, and laminin can be detected by IHC antibodies (Kajbafzadeh et al., 2019).

### 4.6 Mechanical Tests

Following verification of complete cell removal, the impact of decellularization on the mechanical characteristics of ECM is also important. The ECM is composed of a network of molecules that confer a proper mechanical architecture in the tissue required to grow the desired cell population in tissue remodeling. It has been demonstrated that ECM elasticity plays a role in determining stem cell lineage specification, as matrices with elasticity similar to the brain, muscle, or collagenous bone induced neurogenic, myogenic, and osteogenic differentiation of mesenchymal stem cells (MSCs) (Engler et al., 2006). Atomic force microscopy (AFM), and single or bi-axial mechanical tests are vastly used to measure and compare the mechanical strength of the decellularized and native tissue. In AFM, samples are subjected to a probe, and indentation stress curves are obtained. Then these curves are analyzed, and AFC is measured and compared to native tissue (Peng et al., 2019). Moreover, mechanical testing, namely the burst pressure test, can provide insight into the existence, distribution, and integrity of collagen and elastin fibers within dECM (Bielli et al., 2018; Cai et al., 2019).

### 4.7 Additional Tests

Based on the purpose of the study and future function of the grafted dECM, researchers evaluate various components and characteristics of their acellular scaffold. Proteomic analysis of the dECM can precisely show and compare the proteome of the ECM before and after decellularization treatment. Proteomic evaluation can be exerted by different techniques, such as nano liquid chromatography and tandem mass spectrometry, and is used to assess the complete protein profile of the ECM, including remaining enzymes and growth factors (Nakayama et al., 2013). Computed tomography angiography is a technique to demonstrate remained vasculature of tissue after decellularization (Daryabari et al., 2019). In addition, during the process of hydrogel production, the ability of dECM to form gel could be analyzed by gelation assay of dECM to form a gel by gelation assay (Gaetani et al., 2018).

## 5 Pre-Application Processing

**FIGURE 2:**
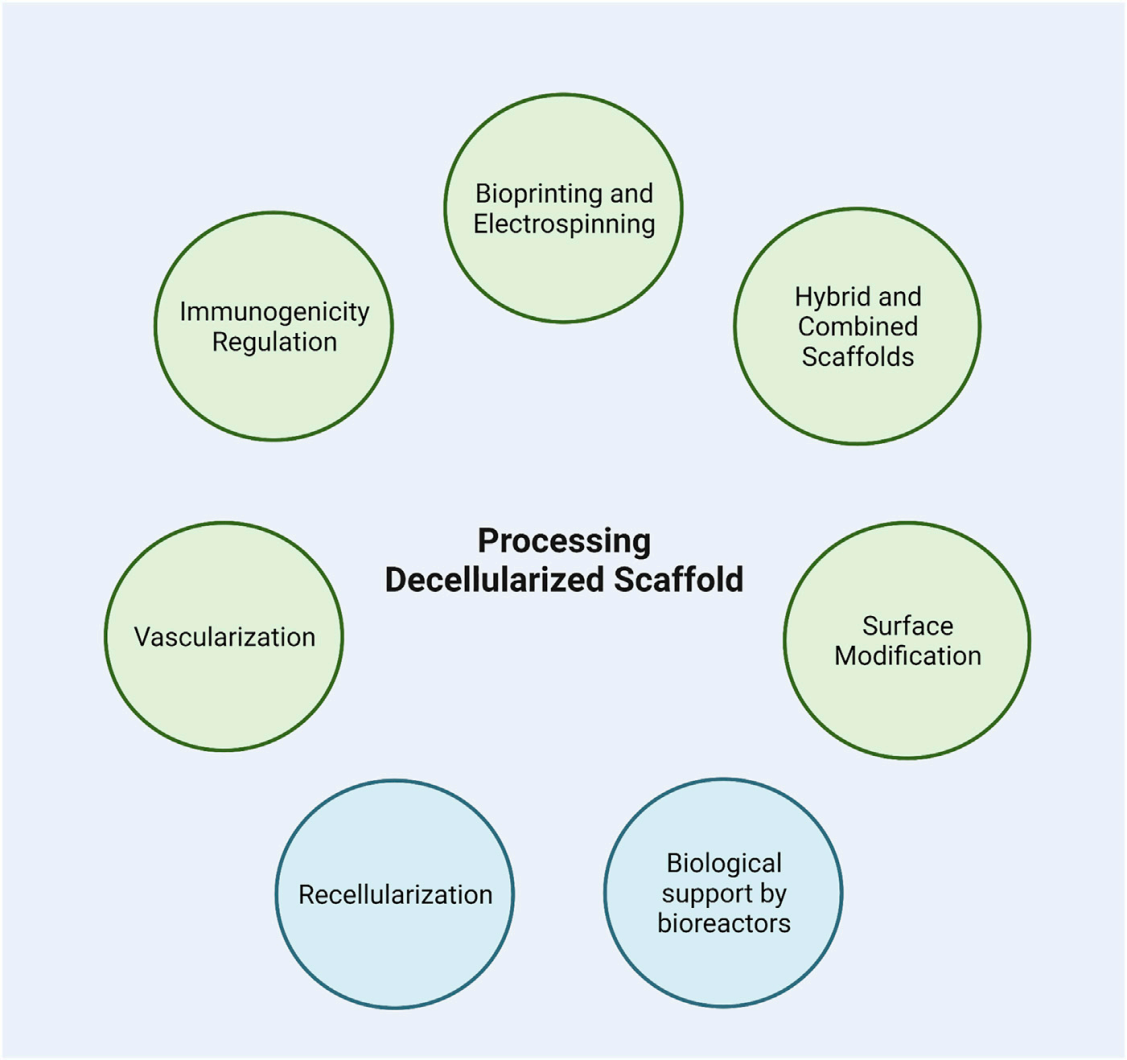
Pre-application processing of decellularized scaffolds. Modifying the decellularized scaffolds improves their capacity to regenerate damaged tissues without causing adverse events. In addition, processing scaffolds with stem cells and bioreactors helps improve their regeneration capacity.

Before being applied, an acellular tissue may undergo a series of *in vitro* procedures, including cell seeding. Processing the scaffolds enhance their ability in successful grafting and function (García-Gareta et al., 2020; Mendibil et al., 2020) (Figure 2).

### 5.1 Modification and Refinement

#### 5.1.1 Vascularization

Oxygen delivery to the tissues is one of the hindrances against applying the decellularized scaffolds, and tissue thickness is an important index for this matter. Preserving the ECM components and vascular structure of the tissue yields better perfusion post-implantation, facilitating further angiogenesis. For instance, laminin and fibronectin in the basement membrane and lamina propria play an essential role in the revascularization of the tissue. Hence, evaluation of the scaffold components and structure after the decellularization process can help us estimate the extent of blood supply (Partington et al., 2013; García-Gareta et al., 2020). Vascularization can be induced by adding angiogenic factors to the matrix or prevascularization of the matrix (Amirsadeghi et al., 2020). Various growth factors were introduced to enhance angiogenesis. Drew et al. observed neovascularization in the presence of exogenous Vascular Endothelial Growth Factor (VEGF) (Dew et al., 2016). However, another study revealed that in bladder tissue regeneration, poly (octamethylene citrate) (POC) composites outperformed small intestinal submucosa (SIS) scaffolds in tissue vascularization, while POC scaffolds lack any exogenous growth factor (Bury et al., 2021). Stem cells are considered beneficial in angiogenesis induction, as they can differentiate into endothelial cells or secrete various growth factors. Adipose-derived stem cells (ADSCs) were reported to significantly enhance vascular network formation in decellularized adipose tissue scaffolds (Han et al., 2015). Prevascularization is a technique of creating microvasculature inside the synthetic scaffold; a decellularized tissue is already prevascularized. Whole organ matrices maintain the internal vasculature of the scaffold, bypassing the perfusion limits regardless of thickness and density (Wang et al., 2019). Hence, it is crucial to produce acellular organs with optimally preserved vessels and ECM composition for repopulating a capable scaffold and restoring blood circulation after implantation.

#### 5.1.2 Immunogenicity Regulation

Non-autologous materials or cells on an engineered organ may trigger the immune response. Activation of the host immune system against synthetic, biological, and decellularized xenogeneic and allogeneic grafts has been reported as a hurdle in the transplantation of engineered tissues and organs (Wiles et al., 2016). Studies have proposed various approaches to overcome immune reaction against engineered tissue, including modulatory coatings on the scaffold surface, immune cloaking, modification of Damage Associated Molecular Pattern (DAMP) proteins which may remain after decellularization, and recellularization with autologous cells to suppress the immune response to materials (Wiles et al., 2016). Moreover, genetically engineered hypoimmunogenic stem cells are alternative to autologous cells to evade immune rejection (Deuse et al., 2019). One of the limitations concerning tissue decellularization is that most studies have examined these scaffolds on animal models, and limited human studies have been conducted so far. Consequently, translation of animal findings on the immune response to humans can be challenging. Human-like animals, such as nonhuman primates and humanized mouse models, with immune systems similar to humans, are being investigated to improve our understanding of the immunogenic components of the acellular scaffold (Bhattacharya et al., 2019; Bilodeau et al., 2020).

#### 5.1.3 Bioprinting and Electrospinning

Bioprinting is a process through which bioinks are used to produce a 3D construct resembling human tissue that can be used to regenerate injured tissues and treat various diseases. Although bioprinting is progressively used in regenerative medicine to develop novel and effective treatments for various diseases, the lack of suitable bioinks hinders its widespread application in medicine (Ferreira et al., 2020; De Santis et al., 2021). Bioinks should be both cytocompatible and appropriate for structuring 3D tissues. 3D bioengineering of tissues using dECM bioink helps provide scaffolds for modeling diseases and developing efficient treatments for them. However, dECM needs augmentation to mechanically resemble the natural ECM (Ferreira et al., 2020; De Santis et al., 2021). Hydrogels could be made through solubilizing dECM followed by reformation of scaffold controlled by temperature and pH. Formation of dECM hydrogels is highly dependent on the collagen content of dECM. Hydrogel bioink derived from dECM can help deliver biomolecules and provide an appropriate surface for cells to migrate and grow (Saldin et al., 2017; Ferreira et al., 2020; De Santis et al., 2021). The biochemical, structural, and viscoelastic properties of dECM are tunable via combination with other biomaterials and crosslinking to provide maximum cytocompatibility along with structural support for tissues and cells. Alginate/dECM compositions as bioink demonstrated beneficial impacts on blood vessel formation after transplantation and prevention of foreign body reactions (Saldin et al., 2017; Ferreira et al., 2020; De Santis et al., 2021). Alginate reinforced with methacrylate-dECM demonstrated augmented bioactivity of hydrogel bioprinted scaffold and osteogenic differentiation of ADSCs (Lee et al., 2020).

Electrospinning has recently become increasingly popular for modifying the dECMs. Electrospinning may help design nanofibrous scaffolds from dECM with similar characteristics to the native tissue, including ultrastructure, porosity, and mechanical characteristics. Electrospun scaffolds demonstrated superior functionality and histocompatibility compared to scaffolds that are combined with other methods. Reinforcing rat decellularized vessels with Polycaprolactone (PCL) using the electrospinning method resulted in increased biomechanical endurance. Electrospinning provides an efficient method to combine natural and synthetic materials to achieve hybrid scaffolds that resemble native tissue with biomimetic architecture and enhanced biomechanical properties (Schenke-Layland et al., 2009; Kai et al., 2013; Gong et al., 2016).

#### 5.1.4 Hybrid and Combined Scaffolds

Combining the decellularized scaffolds with other molecules, including biomaterials, drugs, and growth factors may help modify the characteristics of the decellularized scaffolds. The methods for designing combined scaffolds include solvent casting and particulate leaching, lyophilization (freeze-drying), thermal-induced phase separation (TIPS), gas foaming, rapid prototyping, stereolithography, fused deposition modeling, selective laser sintering, 3D printing, bioprinting, cross-linking, and electrospinning (Eltom et al., 2019; Wasyłeczko et al., 2020).

It was demonstrated that decellularized omentum scaffolds coated with gold (Au) nanoparticles (NP) could provide proper electrical conductivity and characteristics. Hence after seeding cardiac cells, the AuNP-decellularized scaffold can cause higher contraction force, decrease the excitability threshold, and increase the calcium channels currents. Also, silver (Ag) NPs show proangiogenic properties and enhance the biocompatibility of the scaffolds (Shevach et al., 2014; Saleh et al., 2019). When modified with the methoxy polyethylene glycol, an acellular adipose matrix decreases the immunogenicity and increases the adipogenicity of the scaffold (Liu et al., 2021a). Furthermore, polypropylene mesh coated with the ECM inhibits the M1 macrophages and increases the M1/M2 ratio (Wolf et al., 2014). Electrospun PCL blended with collagen in combination with decellularized rabbit aorta was found to provide biocompatibility and rigidity (Ghorbani et al., 2017). Scaffolds fabricated from dECM mixed with PCL have suitable properties for the growth and migration of stem cells. In addition, PCL/Poly Lactic-co-Glycolic Acid (PLGA) electrospun scaffold can support the cell-derived ECM to produce a dECM with desirable mechanical properties and biocompatibility (Schenke-Layland et al., 2009; Bracaglia and Fisher, 2015). Sugar-induced modification of the decellularized kidneys increases mechanical strength and resistance to deformation (Sant et al., 2021). Recently multilayered decellularized scaffolds demonstrated promising bioactivity and should be further studied to be widely used (Smith et al., 2022). Moreover, growth factors and drugs could be loaded on the dECMs, making them more potent. The addition of neurotrophic factors to dECM as well as removing chondroitin sulfate proteoglycans from dECM promote neurite outgrowth (Boyer et al., 2015; Qiu et al., 2020). Heparinized decellularized scaffolds have been examined for promoting angiogenesis and preventing clot formation (Wu et al., 2016). Layer-by-layer coating of the decellularized porcine aortic valve with basic fibroblast growth factor (bFGF) and heparin preserve a sustained release of these factors leading to improved biological activity (De Cock et al., 2010). Another study loaded fibroblast-derived ECM with VEGF and heparin to boost angiogenesis. Application of heparin/VEGF ECM encapsulated with alginate resulted in a prolonged release of these factors as well as enhanced bioactivity (Du et al., 2014). The dECMs are promising options for drug delivery; meanwhile, further studies are needed to increase their effectiveness.

#### 5.1.5 Surface Modification

In order to achieve improved cytocompatibility, mechanical properties, and biological function of decellularized scaffolds without inflammatory reactions, the surface modification could be done via various methods and biomaterials (Ozasa et al., 2013; Liu et al., 2021b). It is demonstrated that immersion of decellularized tendons with carbodiimide-derivatized hyaluronic acid and gelatin could alleviate the tendon’s gliding resistance by augmenting its surface’s smoothness (Ozasa et al., 2013). Moreover, riboflavin-mediated UV crosslinking may repair the damages caused by the decellularization process and increase the smoothness and mechanical strength of the dECM; therefore, it could be used as the luminal surface in the vascular prosthesis (Schneider et al., 2020). Laser micro-ablation is another method that can produce microporosity in the scaffold’s surface (Matuska and McFetridge, 2018). In addition, coating the surface of the scaffolds using heparin reduces the thrombogenicity and makes their surface appropriate to be used as vascular grafts (Wu et al., 2016). Decellularized aorta covalently linked with heparin through “click” coating inhibited platelet adhesion and thrombogenicity. Also, Proper adhesion and proliferation of the endothelial cells were observed (Dimitrievska et al., 2015). Immobilization of VEGF on the surface of dECM through “click” reactions improves angiogenic properties of scaffolds (Wang et al., 2014). Accordingly, surface modification can optimize dECMs for various applications.

### 5.2 Recellularization

Acellular scaffolds have the capacity of *in vitro* or *in vivo* cell seeding. *In situ* tissue regeneration takes advantage of the recipient’s body’s regenerative capabilities and biological supply. Therefore, cell seeding of the scaffold prior to transplantation is not always necessary, but adding the bioactive molecules would reinforce the regeneration process (Yang et al., 2020). Thrombogenic and immunogenic factors on decellularized matrices may trigger thrombogenesis or the host immune response. These factors can be hidden, degraded, or modified by cells. Robertson et al. have shown that reendothelialization of whole heart ECM reduced the scaffold thrombogenicity (Robertson et al., 2014).

During the recellularization process, whole organ repopulation is difficult due to the complexity of the scaffold, the challenges of introducing cells to different parts of the scaffold, the need for the presence of different cell types, and uncertainty about the viability and functionality of cells after organ grafting (Bilodeau et al., 2020). However, advances in cell seeding techniques have provided solutions. Several strategies for the recellularization of a whole organ have been advocated, including slicing, perfusion, and injections (Figliuzzi et al., 2017). For instance, studies reported that the perfusion-based method is fruitful for the recellularization of acellular lungs and kidneys through the trachea and ureter, respectively (Song et al., 2013; Kuevda et al., 2016). Daryabari et al. implanted patches of the decellularized ovine whole uterus into uterine horns of female rats. They demonstrated regeneration of endometrium and myometrium layers, and vascularization was apparent (Daryabari et al., 2019). Whole organ recellularization through the vascular system on the liver, heart, pancreas, kidney, and lung with preservation of original architecture has been widely investigated (Gilpin et al., 2014a; Sabetkish et al., 2015; Scarrit et al., 2015; Ferng et al., 2017). Also, innovative techniques such as the exertion of negative trans-renal pressure gradient and magnetic guidance to direct cells have been proposed for repopulating complex tissues (Song et al., 2013; Ghodsizad et al., 2014).

Moreover, as an alternative method, intraparenchymal injection of the renal cell into the acellular porcine kidneys resulted in satisfactory repopulation and function (Abolbashari et al., 2016). Despite the progress in reseeding methods, slicing the organ demolishes its native 3D structure, the introduction of non-epithelial cells via perfusion is ineffective, and numerous cell injections may cause injuries to the scaffold (Figliuzzi et al., 2017). Therefore, multiple recellularization techniques might be employed to achieve optimal results in the meantime.

Although recellularization is considered a beneficial stage in the regeneration process, *in vitro* cell reseeding could be associated with some constraints. The recellularization process is time-consuming, especially when using induced pluripotent stem cells (iPSCs) derived from the patient’s somatic cells. One of the most important things to keep in mind is to utilize an adequate amount of the appropriate cell type. This factor varies in different tissues and organs and should be considered to reduce the risk of teratoma formation or immune response induction (Badylak et al., 2011; Kim et al., 2020). Multiple types of cells have been investigated for the recellularization of the scaffolds. Limited differentiation and expansion capacity of specialized cells restrains their effectiveness in recellularizing whole organ scaffolds. Stem cells have higher proliferation capacity and are mainly classified into adult stem cells, embryonic stem cells (ESCs), fetal stem cells, iPSCs, and other engineered cells (Denstedt and Atala, 2009; Bacakova et al., 2018). Mesenchymal stem cells (MSCs) and hematopoietic stem cells (HSCs) are the most common stem cells that have been used in the recellularization process. ESCs are pluripotent, but supply issues and ethical concerns restrain their application potential. The iPSCs are produced by the genetic reprogramming of adult somatic cells (Moser and Ott, 2014). Although iPSCs ensure histocompatibility and avoid ethical conflicts, tumor formation remains unresolved. Recently, iPSCs have been shown to produce self-organizing organoids with different cell types through genetic reprogramming or exposure to environmental signaling factors (Ebrahimkhani and Levin, 2021). Gilpin et al. evaluated the recellularization of decellularized lung scaffold with lung endothelial and epithelial progenitor cells derived from human iPSCs. They found decellularized lung scaffold could provide an appropriate environment for iPSC-derived progenitor cells (Gilpin et al., 2014b). Therefore, iPSCs seem promising in providing various functional cells for repopulating whole organs. MSCs are multipotent stem cells and can be easily harvested from various tissues such as bone marrow and adipose tissue. They can differentiate into several cell lineages and have demonstrated encouraging results in recellularizing different tissues. Thus, these cells have been used as a source for reseeding diverse tissues, such as cartilage (Zheng et al., 2011), respiratory tract (Hou et al., 2011; Mendez et al., 2014), urinary tract (Huang et al., 2007; Coutu et al., 2014), and cardiovascular system (Zhao et al., 2010).

It is preferred to use autologous cells for recellularizing acellular scaffolds since these cells would not trigger the host immune system reactions and rejection after transplantation. It has been demonstrated that reseeding acellular diaphragm and lung matrices with stromal cells from homogenized rat lung and diaphragm tissues had lower inflammation and fibrosis formation compared to reseeding with mesenchymal stem cells (Kuevda et al., 2019). In another study by Hellström and colleagues, primary uterine cells and bone marrow-derived mesenchymal stem cells (BM-MSCs) were seeded on rat decellularized uterine scaffolds (Hellström et al., 2016). They observed that these matrices were able to bear pregnancy and normal fetus development. The co-culture of parenchymal cells with non-parenchymal cells could enhance tissue development. Combining tissue-derived cells with endothelial cells or fibroblasts has been reported to improve ECM remodeling, cellular function, and cell organization (Badylak et al., 2011; Gilpin and Yang, 2017). Shen and colleagues have shown that endothelial cells are a critical component for neurogenesis of neural stem cells (Shen et al., 2004). Endothelial cells work as a barrier to prevent thrombosis and organ loss due to immunogenicity of the matrix after transplantation, especially in vascular and valvular grafts (Lichtenberg et al., 2006; Robertson et al., 2014). Furthermore, reendothelialization of whole decellularized heart vascular structures with rat aortic endothelial cells enhanced the contractility of left ventricular constructs (Robertson et al., 2014).

### 5.3 Bioreactors

Intercellular interactions and signaling play a major role in tissue development and function. Bioreactors provide a refreshing environment regarding factors, nutrients, and mechanical force that together enhance cell seeding (Martin et al., 2004). Mostly, bioreactors are perfusion-based systems involving a simple flow. Enhancement in the systems includes gravity involvement or providing a rotatory environment, optimization in oxygenation, or regulation of mechanical stimuli to mimic a natural microenvironment, such as compression and shear stress (Selden and Fuller, 2018). Spinning flasks, rotating cylindrical devices, perfusion bioreactors, and microfluidic systems are among the most used types of bioreactors (Ahmed et al., 2019). Unlike static culture conditions, bioreactors are able to monitor and control the environmental factors precisely. The incorporation of sensors into these devices allows detection of any changes, namely pH or concentration of factors (Simmons et al., 2017). The automation of these systems not only eliminates the need for manual and invasive balancing of the ECM and cellular interactions but also decreases manufacturing costs and enables inclusive clinical application (Martin et al., 2004).

## 6 Application of Decellularized Scaffolds in Regenerative Medicine

### 6.1 *In Vitro* Application

Decellularized scaffolds are being used for modeling the diseases to investigate their pathophysiology. Cell-cell communication, intracellular signaling, stem cell secretions, and therapeutics approaches could be studied in a 3D *ex vivo* scaffold to evaluate the role of the microenvironment in the progression of diseases, such as tumors and inflammatory conditions (García-Gareta et al., 2020). It has been shown that breast cancer cells had improved growth profiles when seeded on decellularized human adipose tissue compared to two-dimensional and Matrigel three-dimensional cultures (Dunne et al., 2014). In a recent study by Wishart and colleagues, the involvement of collagen-IV in breast cancer cell invasion was demonstrated (Wishart et al., 2020). Moreover, using decellularized human colorectal cancer matrices, Pinto et al. advocated that tumoral ECM macrophages enhanced cancer cell invasion *via* cytokine signaling (Pinto et al., 2017).

The underlying mechanism of the diseases and the effectiveness of the drugs could be assessed by modeling the diseases using decellularized scaffolds (McCrary et al., 2020a). For instance, acellular human intestine ECM was cultured with intestinal myofibroblasts to assess intestine fibrosis subsequent to inflammatory bowel disease (Giuffrida et al., 2019). Altered sensitivity of breast cancer cells to doxorubicin and lapatinib was also demonstrated in human adipose tissue-derived ECM scaffolds compared with 2D-cultured cells (Dunne et al., 2014). In another research, higher resistance to drug administration in cancer cells was reported in 3D cultures rather than 2D (Ganjibakhsh et al., 2019). Such studies support the beneficial aspects of *in vitro* application of decellularized tissue from normal or diseased tissues.

### 6.2 *In Vivo* Application

Since decellularized materials are biocompatible and stable compared to synthetic matrices, these cell-free scaffolds are being widely used (Kawecki et al., 2018). However, the attraction of desired stem cells to the site of the scaffold transplantation still remained challenging. Growth factors and chemoattractant substances could help overcome this pitfall (Yang et al., 2020). Sabetkish et al. used decellularized human testicles from patients with testicular feminization syndrome and implanted them between the thigh muscles of mice. Spermatogonial stem-like cells were observed during follow-up (Sabetkish et al., 2021).

**FIGURE 3:**
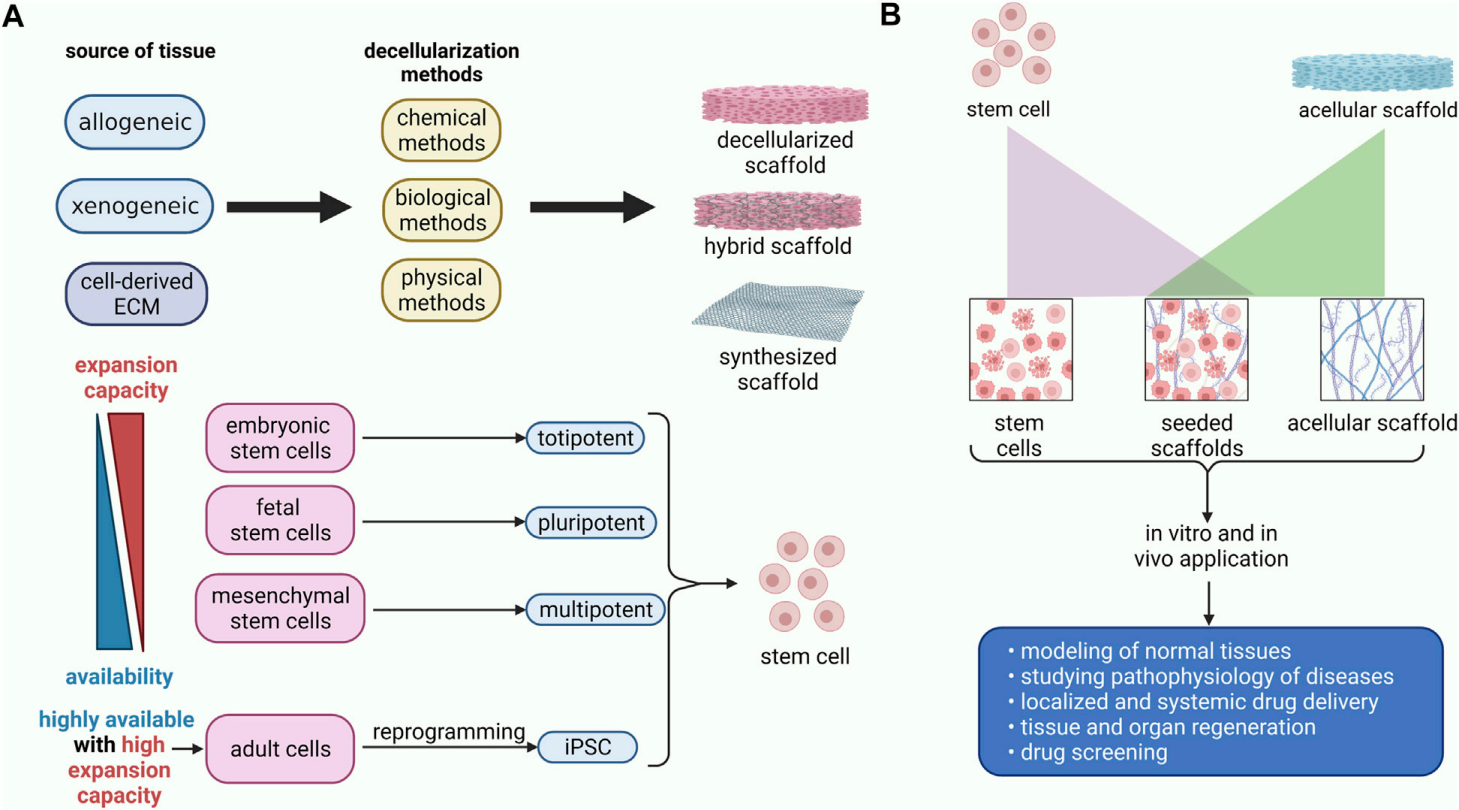
Schematic diagram of preparation and application of the recellularized scaffolds. **(A)** The decellularized scaffolds could be obtained via chemical, biological, and physical approaches from human or animal sources. It has been demonstrated that stem cells from embryonic sources have a higher expansion capacity, albeit they are not easily accessible. Conversely, stem cells from adult sources, including mesenchymal stem cells, are highly available, whereas they do not have proliferation and differentiation capability as much as embryonic stem cells. Nevertheless, mesenchymal stem cells are multipotent stem cells that can differentiate into various cell lineages. The iPSC can be obtained via genetic modification of mature cells. Thus, they are highly expansible and readily available. **(B)** decellularized scaffolds can be used as cell-free grafts, or they can be seeded with stem cells. Decellularized scaffolds with or without stem cells could be transplanted for regeneration of the damaged tissues. Moreover, they are used in drug delivery, drug screening, disease modeling, and studying the pathophysiology of diseases.

Decellularized tissues, either cell-free or recellularized, primarily aim to function as implantable matrices for lost or injured organ regeneration (Figure 3). Multiple tissues have been introduced as candidates for tissue engineering, including the gastrointestinal tract, respiratory system, vascular, and neural tissues (Mendibil et al., 2020). Gilpin et al. have classified the applicable acellular scaffolds into cell sheets, tissues, and whole organs (Gilpin and Yang, 2017). Cell sheets are simple constructs mainly derived from a single cell type and may heal minor lesions or more complex structures when different sheets are combined. Cell-derived matrices are state-of-the-art ECM that can be used as an alternative to native tissue-derived matrices since human cells are accessible and limitless, and matrix properties can be controlled (Fitzpatrick and McDevitt, 2015).

It is evidenced that genetic modification may improve the characteristics of ECM and the efficacy of the decellularization process. Effective decellularization process could be reached via apoptosis of death-inducible stem cells. Also, genetic modification of the stem cells may help tune the ECM composition, such as modifying VEGF expression by stem cells (Papadimitropoulos et al., 2015; Bourgine et al., 2017; Morris et al., 2018). Acellular ECM derived from BM-MSC sheets was used to treat osteochondral defects in rabbits (Wang et al., 2020). These matrices promoted regeneration of articular cartilage and subchondral bone with some degrees of vascularization. Reconstruction of blood vessels, combination of various decellularized cartilage sheets to produce two-dimensional cartilage assembly, and recellularization of periodontal ligament cell sheets were also reported (Fitzpatrick et al., 2008; Gilpin and Yang, 2017).

Acellular ECM hydrogels can also provide a scaffold for transplantation and injection (Young et al., 2011; Wolf et al., 2012). In addition, transplantation of recellularized acellular scaffolds has been approached by many researchers. In a study, regenerated rat lungs were orthotopically transplanted to investigate the function of reendothelialized pulmonary vasculature (Tsuchiya et al., 2017). They showed blood perfusion in the transplanted lung as well as leak-free ventilation. Transplantation of reseeded decellularized diaphragm into rats resulted in improved spirometry parameters (Gubareva et al., 2016).

Currently, decellularized tissues are widely being studied for organ regeneration. Kajbafzadeh and colleagues have implanted acellular rat colon-derived scaffolds into the mesenteric tissue as a graft with an end-to-end anastomosis to the colon of host rats that resulted in recellularization of the scaffold (Kajbafzadeh et al., 2020). The trachea, heart valve, and urethra are examples that have been clinically regenerated with decellularized scaffolds (Versteegden et al., 2017; Porzionato et al., 2018).

### 6.3 Human Studies

Human application of different acellular organs is probably the ultimate goal of tissue engineering and regenerative medicine. Due to the shortcoming of living organs donation, decellularization and tissue engineering methods could pave the way for regenerating tissues and organs to treat diseases in a less invasive procedure.

Variability in human tissues and their immunogenicity makes it inconceivable to propose a standardized decellularization method, thus precluding acellular whole-organ transplantation (Tenreiro et al., 2021; Mattei et al., 2017). Other hindrances include ideal vascularization, innervation, or recellularization of acellular scaffolds (Choudhury et al., 2018). Application of cell-free grafts is a relatively new area of the clinical setting to use acellular matrices. Many commercial companies have been producing dECM, namely Biohorizon and Axogen, which produce acellular skin graft and dECM nerve scaffolds. In addition, many human organ-derived dECM-bioprinted hydrogels have been used in drug screening and delivery (Choudhury et al., 2020; Lopresti et al., 2015; Cui et al., 2019). Also, AlloDerm (derived from the allogenic human cadaveric dermis), Strattice (derived from porcine dermis), and OaSIS (derived from porcine SIS) are commercial cell-free products that have been used in recent decades mainly for covering the skin flaps donor sites, breast reconstruction, and managing ulcers and wounds, respectively (Cui et al., 2019). In a clinical trial, human acellular vessels showed safety and functionality in providing access for hemodialysis in end-stage renal disease patients (Lawson et al., 2016). Different decellularized tissues and their application status is reviewed by Liao et al. (Liao et al., 2020). Currently, several clinical trials in different phases are investigating the application of decellularized tissue in the regeneration of injured or lost organs. An active phase II study is being conducted at the Department of Biomedical Engineering, Johns Hopkins University, investigating acellular adipose tissue for soft tissue reconstruction (identifier: NCT03544632, clinicaltrials.gov). Various decellularized scaffolds from animal and human sources have been used clinically, mainly for wound healing and surgical mesh devices, albeit their application is still limited, and further studies are needed to make decellularized scaffolds commercially available treatment of disease (Damodaran and Vermette, 2018).

## 7 Discussion: Challenges and Future Directions

**FIGURE 4:**
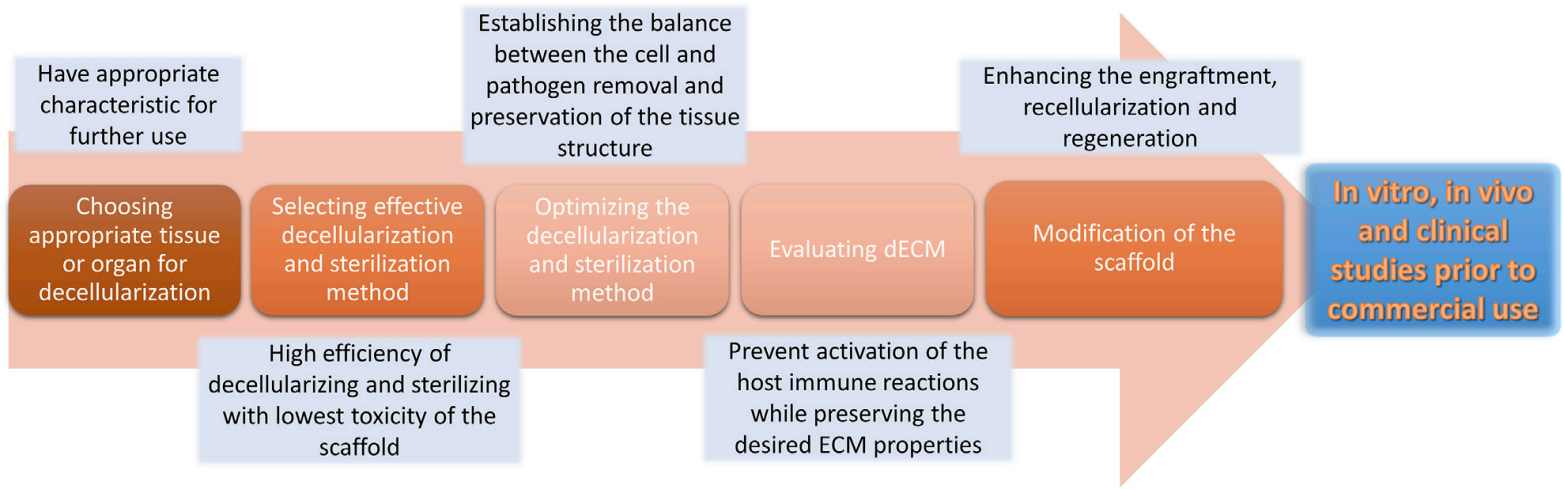
Challenges and hurdles regarding the decellularization process. The decellularization process encounters different challenges that may affect the resulting scaffold.

Despite favorable characteristics of the decellularized scaffolds for implementation in clinical practice, there are some constraints and challenges in their application. Determining the suitable tissue, decellularization and sterilization method selection, optimizing the efficacy of these techniques, dECM quality assessment, modification of acellular scaffolds *in vitro* and *in vivo* study, and transplantation problems (e.g., the timing of biomaterial transplantation) are among the unsolved challenges regarding the clinical translation of the decellularized scaffolds (Figure 4) (Zhang et al., 2021).

The decellularization method is selected based on the type of tissue and its characteristics. Developing cell removal techniques with lower toxic agents or productions, in addition to enhancing sterilizing techniques, would be beneficial. Apart from that, unlike most synthetic scaffolds (Han et al., 2015), purified ECM provides a natural microenvironment for the cells that is challenging to preserve its properties during the decellularization process (Frantz et al., 2010; Crapo et al., 2011). Ongoing research is aspiring to develop new techniques to better preserve the ECM architecture and components, such as vacuum-assisted and apoptosis-assisted decellularization (Zhang et al., 2021). Removing cells from ECM can become more efficient if an innate cellular process or an exogenous stimulus, such as a drug, promotes cell death, thereby eliminating possible ECM structural damage. Cartilage ECM was yielded by culturing human mesenchymal stromal cells with death-inducible genetic systems. This method resulted in more productive bone tissue engineering compared to cartilage ECM obtained from freeze-thaw cycles (Bourgine et al., 2014). Also, camptothecin-treated nerve tissue resulted in cellular apoptosis and clearance, which was immunogenically tolerable *in vivo* (Cornelison et al., 2018).

Various evaluation tests have already been established to ensure that cellular compounds have been cleared from the dECM during the decellularization treatment, while the chemical and biomechanical properties remain intact and the residual detergent is removed from the decellularized tissue, as the remaining detergents may cause cell toxicity in the scaffold (Zhang et al., 2021). Each tissue has unique characteristics that make it proper for a research plan and further clinical use. Additional novel analyzing tests should be developed for each tissue and its future point of function, letting researchers assess the acellular scaffold specifically and exclusively.

Application of naturally-derived ECM is restricted owing to limited animal sources that are free of by-products and contaminants, while synthetic scaffolds are available in ample amounts (Frantz et al., 2010). Polymerization of biodegradable materials is becoming increasingly popular since manufacturing techniques such as electrospinning can help create a large number of scaffolds with controllable indexes, including shapes, porosities, and fiber arrangements (Nam et al., 2007; Langer and Vacanti, 2016; Asghari et al., 2017). In order to overcome the damages caused by the decellularization process and take advantage of engineered materials, many researchers have attempted to modify decellularized scaffolds, print 3D scaffolds using bio-inks, or design ECM-derived hydrogels (García-Gareta et al., 2020). In terms of regenerative medicine, 3D printing is a valuable tissue fabrication technology since it is capable of reproducing the structure of the desired organ; however, clinical application of these 3D bioprinted constructs depends on further regulations and improvements (Ji and Guvendiren, 2017; Bilodeau et al., 2020).

Although the aforementioned strategies have been effective to a large extent in the decellularization process, there are still many hindrances in the endless road of dECM engineering. Despite all progress in this field, further investigations are needed to overcome challenges regarding the clinical application of decellularized biomaterials and the ultimate goal of whole organ transplantation.

## Glossary

ECM: extracellular matrix
dECM: decellularized extracellular matrix
3D: three-dimensional
GAG: glycosaminoglycans
SDS: sodium dodecyl sulfate
SD: sodium deoxycholate
SB: sulfobetaine
TnBP: tri (n-butyl) phosphate
CHAPS: 3-[(3-cholamidopropyl)dimethylammonio]-1-propanesulfonate
DNase: deoxyribonuclease
EGTA: ethylene glycol tetraacetic acid
EDTA: ethylenediaminetetraacetic acid
PAA: peracetic acid
RNase: ribonuclease
UV: ultraviolet
MTT: 3-(4, 5-dimethylthiazol-2-yl)-2, 5-diphenyltetrazolium bromide
dsDNA: double-stranded DNA
H&E: hematoxylin and eosin
DAPI: 4’,6-diamidino-2-phenylindole
SEM: scanning electron microscope
TEM: transmission electron microscopy
IHC: immunohistochemistry
MSCs: mesenchymal stem cells
AFM: atomic force microscopy
VEGF: vascular endothelial growth factor
POC: poly(octamethylene citrate)
SIS: small intestinal submucosa
ADSCs: adipose-derived stem cells
DAMP: damage associated molecular pattern
TIPS: thermal-induced phase separation
Au: gold
NP: nanoparticle
Ag: silver
PLGA: poly lactic-co-glycolic acid
bFGF: basic fibroblast growth factor
iPSCs: induced pluripotent stem cells
ESCs: embryonic stem cells
HSCs: hematopoietic stem cells
BM-MSCs: bone marrow-derived mesenchymal stem cells
